# Effects of Blended Simulation on Nursing Students’ Critical Thinking Skills: A Quantitative Study

**DOI:** 10.1177/23779608231177566

**Published:** 2023-05-18

**Authors:** Anders Sterner, Robert Sköld, Henrik Andersson

**Affiliations:** 1 101066 Faculty of Caring Sciences, Work Life and Social Welfare, University of Borås, Borås, Sweden; 2 Centre for Prehospital Research, Faculty of Caring Science, Work Life and Social Welfare, University of Borås, Borås, Sweden; 3 Centre of Interprofessional Cooperation within Emergency Care (CICE), Linnaeus University, Växjö, Sweden; 4 Department of Health and Caring Sciences, Linnaeus University, Växjö, Sweden

**Keywords:** simulation training, education, blended simulation, critical thinking, nursing education research

## Abstract

**Introduction:**

Critical thinking is regarded as imperative to healthcare quality and patient outcomes; therefore, effective strategies in nursing education are required to promote students’ critical thinking abilities, leading to their success in clinical work. Accordingly, simulation-based education has been suggested as a measure for achieving this goal.

**Objective:**

The aim of this study was to explore whether a nursing education course with blended simulation activities (hands-on simulations with high-fidelity manikins and a web-based interactive simulation program) could increase nursing students’ critical thinking skills.

**Method:**

A quasiexperimental, one-group pretest and post-test design was utilized. Data were collected through premeasurement and postmeasurement using a critical thinking questionnaire and were analyzed using paired sample *t*-tests, independent sample *t*-tests, and the nonparametric Wilcoxon signed-rank test. The effect size was calculated using Cohen's *d* formula.

**Results:**

Sixty-one nursing students (57 women and four men, mean age 30 years) participated in the study. Findings of the paired sample *t*-test showed a significantly higher mean score for posteducation than pre-education, indicating a significant change in nurses’ critical thinking capabilities (*p* < .001). The results for Cohen's *d* formula ( − 0.87) of the mean scores between pre-education and posteducation indicated a large effect size. The Wilcoxon signed-rank test also showed a statistically significant increase in the students’ critical thinking abilities between pre-education and posteducation measures (*p* < .001). No statistically significant differences were found in the mean score according to age or sex.

**Conclusion:**

This study concluded that blended simulation-based education can increase nursing students’ critical thinking capabilities. As a result, this study builds on the use of simulation as a measure for developing and promoting critical thinking abilities during nursing education.

## Introduction

The concept of critical thinking has roots dating back to the work of Socrates. Today, nurse educators often refer to critical thinking as a part of nurses’ everyday work and thereby as an important component of their success in clinical work. Hence, nurse educators need to foster and encourage critical thinking in complex care situations (Von Colln-Appling & Giuliano, 2017). For decades, nurse educators have struggled to find and use methods for facilitating and evaluating critical thinking skills (Von Colln-Appling & Giuliano, 2017). These approaches need to be student-centered, involve cooperation between students as well as with educators, and take place in an open atmosphere and student-active educational environment (Westerdahl et al., 2022). Carvalho et al. (2017) explain that one of the most used education strategies for promoting critical thinking in nursing education is problem-based learning (PBL). Other strategies include concept mapping, tutoring, reflective writing, and simulation training. Simulation-based education is recommended in nursing education by the World Health Organization (2018), with the argument that one benefit of this method is the improvement of students’ critical thinking in complex care situations.

## Review of Literature

Critical thinking is integrated into numerous clinical assignments and responsibilities faced in nursing (Westerdahl et al., 2022), and it is considered crucial to the quality of healthcare and patient outcomes in acute care settings (Willers et al., 2021) in which nurses must be able to think quickly and anticipate outcomes in a matter of seconds because it could mean the difference between life or death to a patient (Von Colln-Appling & Giuliano, 2017). As a result, well-developed critical thinking skills are required for nursing (Carter et al., 2015). However, the concept of critical thinking is ambiguous and diverse, and there is no universally accepted conceptual framework in nursing to describe and evaluate critical thinking (Zuriguel Perez et al., 2015). A landmark Delphi study by Facione (1990) described critical thinking as a purposeful self-regulatory judgment that leads to interpretation, analysis, evaluation, and inference. This finding has since been investigated in nursing using the California Critical Thinking Disposition Inventory (CCTDI) (Carter et al., 2015; Noone & Seery, 2018; Sommers, 2018). Another tool for evaluating critical thinking frequently used in nursing literature is the Watson–Glaser Critical Thinking Appraisal (Carter et al., 2015; Zuriguel Perez et al., 2015).

Critical thinking in nursing education begins with nursing students’ capability to acquire knowledge and apply it, for example, in pathophysiology, medical treatment, and nursing actions. However, nursing students also need the capability to interpret and analyze clinical information. This aspect requires the capability to distinguish relevant from irrelevant information and determine whether additional information is necessary (Von Colln-Appling & Giuliano, 2017). To foster, encourage, and maximize students’ critical thinking skills in undergraduate nursing education, various strategies, such as PBL, concept mapping, tutoring, reflective writing, and simulation-based education, have been tested and suggested (Carvalho et al., 2017).

In simulation-based education, learners interact with people, simulators, computers, or task trainers to accomplish different learning goals. The degree to which the simulation replicates the real event is described as fidelity. High-fidelity simulation refers to experiences that are extremely realistic and provides a high level of interactivity and realism for the learners (Lioce et al., 2020). Simulation-based education is a pedagogical method that can also be used by nurse educators to facilitate and evaluate critical skills (Andersson & Sterner, 2022). Simulation-based education can strengthen nursing students’ clinical preparedness while teaching them how to assess, prioritize, and plan patient care (Westerdahl et al., 2022). According to Persaud and Thornton (2018), simulation-based education is also an innovative way to integrate caring behaviors into the nursing curriculum during undergraduate nursing education. In this method, reflection is considered imperative and thus should be included in all simulation-based experiences aiming to improve future performance (International Nursing Association of Clinical and Simulation Learning Standards Committee, 2016).

Research investigating the effectiveness of simulation-based education and the development of critical thinking abilities in nursing education is conflicting. The reasons for inconsistent results in reviews might be due to the low number of included studies and the small sample sizes (Alshehri et al., 2022). However, in a systematic literature review, Adib-Hajbaghery and Sharifi (2017) explored the effects of simulation on nurses’ and nursing students’ critical thinking. They found that, although most studies demonstrated that simulation positively impacts critical thinking, only half of the examined studies reported a statistically significant positive effect. A possible explanation for this inconsistency was the use of different measurement instruments and the wide variation in the simulation methods used. When Li et al. (2022) explored the effectiveness of high-fidelity simulation for Bachelor of Science Nursing Students and found no obvious advantage in developing students’ critical thinking compared to other teaching methods. However, when discussing these results, the authors raised the question of whether the inconsistencies in their findings could also be attributed to differences in the duration of the simulation training. The potential of repeated simulation to enhance critical thinking skills is an important issue to consider when using simulation-based education (Al Gharibi & Arulappan, 2020). Nevertheless, Blakeslee (2020) concluded that, instead of focusing on a single teaching strategy, the current literature encourages nurse educators to develop multiple comprehensive teaching strategies to improve nursing students’ critical thinking skills.

A newly developed approach combining multiple comprehensive teaching strategies and simulation-based education is referred to as “blended simulation.” Blended simulation can be described as combining hands-on simulation, such as the use of high-fidelity manikins, with computer-based simulation in the same course. Exploring this approach with nursing students in the second and third years revealed that this approach addresses curricular objectives in different but complementary ways, thus facilitating personal engagement and reflection and providing relevant clinical–practical learning experiences (Johnsen et al., 2021). To the best of our knowledge, no studies to date have explored the use of a blended simulation approach in facilitating nursing students’ critical thinking. Consequently, the current study aimed to explore whether a nursing education course with blended simulation activities could increase nursing students’ critical thinking skills.

## Methods

### Study Design and Settings

This study adopted a quasiexperimental, one-group pretest and post-test design. The study was performed at a university in the southwest of Sweden, where nursing education is a university-level 3-year program. All graduates receive a Bachelor of Science degree in nursing or caring science.

The present study was summarized and reported inspired by Cheng et al.'s (2016) study “Guidelines for Healthcare Simulation Research: Extensions to the CONSORT and STROBE Statements.”

### Course

The course Caring Assessment and Intervention of Sudden Illness is allocated 7.5 European Credit Transfer System credits. Training in this course emphasizes nurses’ patient care responsibilities in emergency care settings. The course uses a web-based learning management system in which materials, such as curriculum, course-specific documents, web links, and recommended literature, are presented to the students. The course is completed in semester five of six and is held at the university's clinical training center (CTC). The course is one of several obligatory courses in the current nursing education that are implemented in whole or in part at the CTC using simulation in various forms as a didactic strategy. The present course is held at the CTC and includes multiple comprehensive teaching strategies, such as blended simulation activities. Teachers in this course are all nurse specialists, have extensive work experience in emergency care settings, and are trained and experienced in simulation-based education.

### Content of the Course

The course is divided into three segments: patient assessment, teamwork, and nursing and medical guidelines in an emergency. An overview of the course content, objectives, and learning activities is presented in Table 1. The simulation sessions are conducted during the assessment and teamwork segments, following a presentation of simulation guidelines for best practice with prebriefing, briefing, simulation, and debriefing sessions (Watts et al., 2021). The debriefing session is based on reflection and feedback and is conducted in a safe and open environment. The session includes three phases: description, analysis, and application (Hall & Tori, 2017).

**Table 1.** Overview of the Course Content, Objectives and Learning Activities.

Content	The content is on how the nurse can carry out structured nursing assessments, provide care and treatment along with reporting of patients affected by sudden illness or injury.	
Objectives	Knowledge	Describe theoretical foundations for: – nursing assessments and care of patients – structured reporting of patients – structures for safe care of patients
	Skills	Apply theories and structures for: – nursing assessments and care of patients – structured reporting of patients – structures for safe care of patients From a team perspective and based on the patient's living conditions, illnesses and health risks: – plan, implement, evaluate, and document the care activities
	Attitude	Evaluate: – relevant information and knowledge regarding nursing assessment, care, and reporting – one's own approach in connection with nursing assessment, care and reporting based on a patient, relatives, and nurses’ perspective, – own needs for additional knowledge regarding nursing assessments, provide care and treatment along with reporting of patients
Learning activities	Lectures/seminaries	– Prerecorded films(movies) – Litterature – Articles – Interactive simulation system review – Simulation debriefing – Questionnarie – Case presentation seminar
	Simulation	– Interactive A–E structured assessment and reporting simulation – Hands on team simulation according to crisis resource management (CRM) criterias – Interactive simulation according to a apply medical and patient-centered care guidelines.

The patient assessment step focuses on an A–E assessment method and maintaining vital body functions using oxygen therapy and fluids (Thim et al., 2012). Students start this segment of the course by reading the literature and watching prerecorded instructional videos. In addition, they can also reach national guidelines on how to treat patients through oxygen and fluid therapy. The students are also required to answer and submit a question on how to perform an assessment and treat a patient in an emergency.

**Figure 1.**
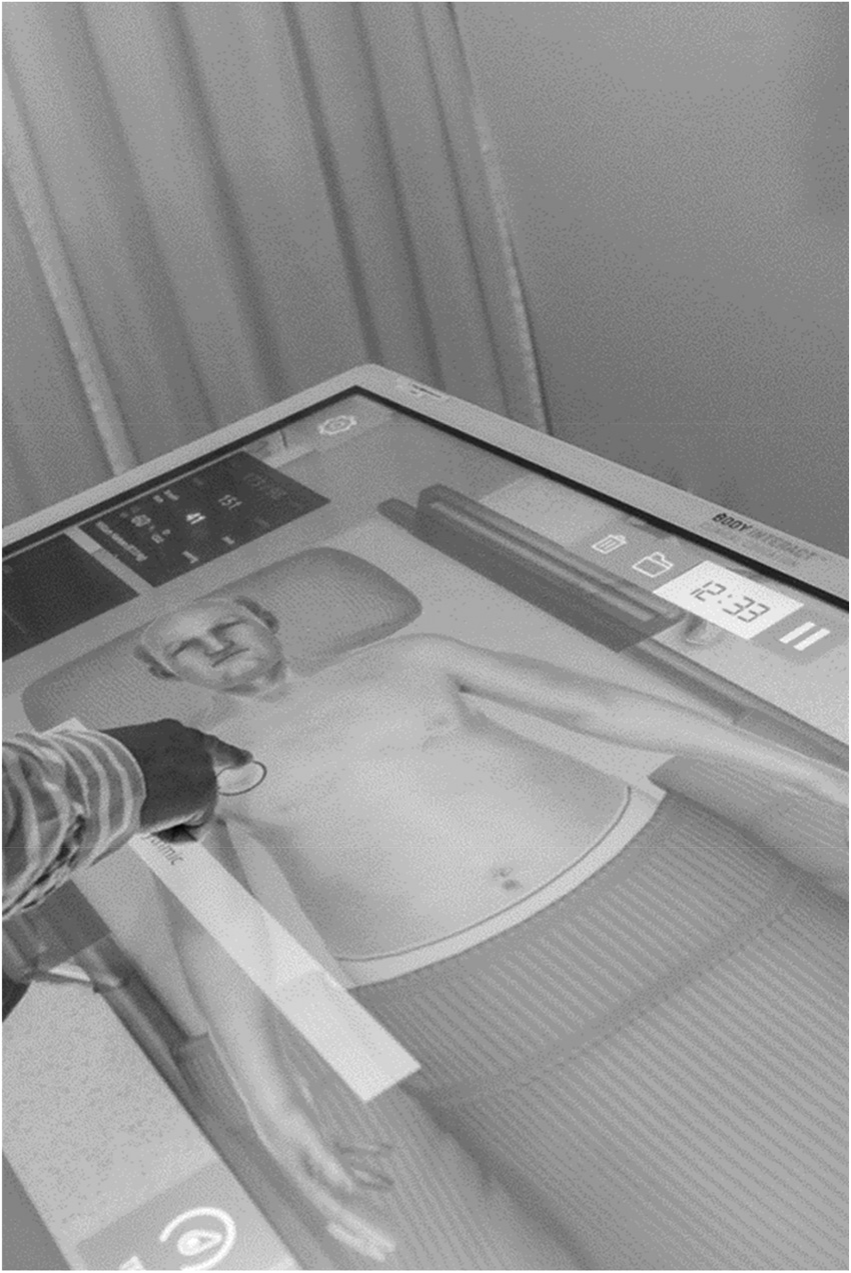
Body interact table.

To develop their assessment skills, the students use a web-based interactive simulation program, Body Interact (BI), as a digital patient simulator. BI is accessible from the web and is also integrated into a 55-inch flat-screen, multitouch horizontal table at CTC (Figure 1).

BI allows students to interact with patients of different genders and ages. They can perform history-taking, conduct a physical examination, monitor vital parameters, request different diagnostic tests, and administer various medications. After a short briefing about how to use the system, the students receive their login details for the system to start their assessment training. These login details give them access to five different cases and allow them to train as frequently as needed. After approximately a week of access to BI, the students arrive at the campus for a BI simulation training session. During the session, they simulate one of the cases, followed by a debriefing. In this session, they receive feedback and have time to reflect on their actions with a teacher and fellow students. After the BI session, they are encouraged to continue refining their assessment skills based on their performance. These skills will be examined subsequently in a simulation in BI, for which the students will be raffled to one of five cases they previously practiced. The students’ performance in the examination is assessed using a summary sheet of the assessment points that must be achieved to pass the examination. This document is presented on a web-based learning management system at the start of the course.

In the teamwork step, the hands-on simulation focuses on crisis resource management (CRM) (Rall & Dieckmann, 2005). Students start this segment of the course by reading the literature and watching prerecorded instructional movies. They are also required to answer and submit a question about how to relate the CRM key points to the simulation. The students are also allowed to test the functions of the simulation manikins and familiarize themselves with the simulation environment. The hands-on simulations take place at a CTC in a room replicating and equipped like a hospital environment. Simulators used in the hands-on simulations are high-fidelity manikins made by Gaumard (HAL s3101 and Pediatric HAL s3005). Before the examination, the students have opportunities to engage in teamwork in groups of three on two occasions. The first training session is a simulation guided by a teacher that includes a debriefing session. The second training session is a peer learning session in which the debriefing session is managed by the students using predefined questions presented on the web-based learning management system. Both simulations focus on the CRM and A–E assessment. The hands-on simulation examination has two components: the students must follow seven specified CRM key points and describe the strengths and weaknesses of their hands-on simulation performance, respectively, to pass. All students are individually examined.

The nursing and medical guideline segment focuses on following evidence-based guidelines. The same five cases previously used in the web-based simulation assessment with BI are used in this section of the course. The students create a presentation to describe the patient's care from the assessment, the pharmacology treatment, and the specified nursing objectives. Numerous objectives are presented on the web-based learning management system and need to be included in the presentation. The students also have access to guidelines associated with the patient's specific diagnosis. After the medical treatment, the students must define the nursing objectives, how to treat the patient, and how to follow up on the treatment given. An examination is performed during a seminar in which the students are raffled to present one of the five cases they have worked with throughout the course.

## Measurement

A critical thinking questionnaire (CTQ) was developed in Swedish based on the CCTDI and the Watson–Glaser Critical Thinking Appraisal (Wilde-Larsson et al., 2018). The CTQ is treated as a unidimensional scale since no underlying dimensions or factors are presented (Boateng et al., 2018). The CTQ consists of 28 items on a 4-point scale, with a total score ranging from 28 to 112 points. The higher the score, the greater the critical thinking capability. During the development of the CTQ, face and content validity were established, as well as acceptable reliability scores, with Cronbach's alpha ranging between 0.91 and 0.66. CTQ has previously been used on nursing students in Sweden, Norway, and Indonesia (Wilde-Larsson et al., 2018).

## Data Collection and Participants

Data were collected from September 2021 to May 2022. All nursing students (*n*  =  144) in six cohorts enrolled in the allocated course were invited by the first author to participate during their course introduction. Oral information about the study was given during the invitation, and a link to the web-based questionnaire was distributed to the allocated learning platform for the course. The link presented additional written information about the study and the contact information of the research group. Additional data collection was performed after the last lecture of the course. The questionnaire was filled out by hand in privacy.

## Analysis

Statistical analysis was performed using SPSS software version 27. Descriptive statistics and frequency statistics were used to analyze missing data, errors, and demographics. Missing data utilized the baseline-observation-carried-forward (BOCF) method. Normal distribution was verified using the Kolmogorov–Smirnov and Shapiro–Wilk tests. Pre–post changes were evaluated using paired sample *t*-tests. Effect sizes for the tests were calculated using Cohen's *d* formula. Effect sizes of 0.2 were considered small, 0.5 was considered medium, and 0.8 was considered large (Cohen, 1992). Internal consistency of the CTQ was assessed using Cronbach's alpha. Differences in demographic variables (sex, age) and pretest and post-test scores were analyzed using independent sample *t*-tests (2-tailed). Due to the ordinal nature of the scale, the nonparametric Wilcoxon signed-rank test was also applied. The level of significance was considered at *p* < .05.

## Ethical Considerations

This type of study does not fall under the national act of the Ethical Review of Research Involving Humans (SFS 2003:460, 2003). However, the study was approved by the faculty of nursing education at the university. The study was also conducted according to the Declaration of Helsinki (World Medical Association, 2013) by ensuring that participation in the study was voluntary and responses were treated with confidentiality. This move was crucial because the study involved teachers in the course. Therefore, AS invited and informed the participants about the study to ensure voluntary participation. AS met the students in the course only during the invitation. The teachers in the course knew which students in a cohort participated in the study only when they collected the post-test data at the end of the course. No data were analyzed before completing the collection process to minimize influencing the students or teachers in the course. Informed consent to participate was assumed if the nursing students completed and submitted the survey.

## Results

In total, 144 nursing students were eligible to participate in this study. Sixty-six (46%) participated and subsequently responded to the CTQ as a pretest measure. At the end of the course, 61 (92%) of these nursing students responded to the CTQ as a post-test measure. Accordingly, an analysis was performed of the data from the 61 (42%) participants. The majority were female (n  =  57 [93%]). The mean age was 30 (SD: 7.54), with a range of 20–50 years. Descriptive statistics revealed no missing data on the pretest, as the electronic questionnaire could not be submitted if any data were missing. However, missing data were noted on the post-test scores of six participants. The missing scores consisted of six different items, one for each participant, indicating that the missing values could be considered random. Therefore, BOCF method was utilized for missing data imputation.

The normal distribution of the pretest score was calculated and assessed using the Kolmogorov–Smirnov test (*p*  =  .200) and Shapiro–Wilk test (*p*  =  .525). Because the data indicated normality, the mean scores of the total score were compared between pre-education and posteducation. The paired sample *t*-test indicated that the mean posteducation score was significantly higher (*p* < .001), indicating a statistically significant change in nurses’ critical thinking capability (Table 2). The effect size of the mean scores between the pre-education and posteducation data was calculated using Cohen's *d* effect size ( − 0.87), indicating a large effect size. As a measure of reliability on the CTQ, internal consistency was assessed using Cronbach's alpha. The CTQ scale of the pre-education and posteducation tests demonstrated alpha coefficients of 0.622 and 0.704, respectively. These results indicate that the internal consistency of the CTQ pre-education was moderate, whereas that of the posteducation was relatively high.

**Table 2.** Paired Sample *t*-test, Cohen's *d* Effect Size and Cronbach's Alpha.

	*n*	Mean	SD	S.E. mean	Paired *t* test			Cohen's *d*	Cronbach's alpha
					*t*	df	*p*			
CTQ Pretest post-test	61 61	84.34 88.67	5.66 6.33	0.725 0.811	−6.81	60	*p* < .001	−0.87	0.622 0.704

SD  =  standard deviation, S.E  =  standard error, *t*  =  *t*-value, df  =  degrees of freedom; *p*  =  two-tailed.

Independent sample *t*-tests showed no statistically significant differences in the pre-education mean score according to age or sex, nor did the posteducation mean score (Table 3). The Wilcoxon signed-rank test also indicated a statistically significant increase in students’ critical thinking abilities between pre-education and posteducation measures (*N*  =  61; *Z*  =  5.267; *p* < .001). The total score results were 47 positive, 10 negative, and four ties (no change).

**Table 3.** Independent *t*-test.

Independent *t*-test							
	Younger *n* *=* 32		Older *n* *=* 29				
	Mean	SD	Mean	SD	df	*t*	*p*
Total score pre-education	84.16	5.48	84.55	5.93	59	−0.270	.788
Total score posteducation	89.41	5.61	87.86	7.05	59	0.950	.346
	Men *n* *=* *4*		Female *n* *=* *57*				
	Mean	SD	Mean	SD	df	*t*	*p*
Total score pre-education	85.75	4.27	84.25	5.76	59	−0.511	.611
Total score posteducation	88.50	5.80	88.68	6.41	59	0.056	.956

SD  =  Standard deviation, df  =  degrees of freedom, *t*  =  *t*-value, *p*  =  two-tailed.

## Discussion

The main finding of this study was that blended simulation-based education had an impact on nursing students’ critical thinking capabilities, and no differences were found in critical thinking capability based on sex or age.

This study indicates that a course with blended simulation-based education increases nursing students’ critical thinking capabilities. The use of blended simulation-based education is a way for educators to expose students to real-world situations without risking patients’ well-being, while introducing caring assessment and interventions for when sudden illness occurs. This result can be seen in light of other simulation research conducted on high-fidelity simulation and computer-based simulation. Research focusing on high-fidelity simulation has indicated that it improves critical thinking skills (Guerrero et al., 2022; Hanshaw & Dickerson, 2020). However, research on virtual simulation has indicated some uncertainty that virtual simulation can improve students’ critical thinking compared to traditional instructional methods, such as lectures, practical lessons, and tutorials (Foronda et al., 2020). This means that the outcomes of students’ learning through blended simulation-based education can be questioned when high-fidelity simulation and computer-based simulation are contrasted.

Simulation is an experiential learning process (Jeffries, 2012), and critical thinking is an important indicator of student learning quality (Alsaleh, 2020). Nurse educators need to create learning situations in which nursing students can transform their theoretical knowledge into various patient-related problems in specific care contexts (Mikkonen et al., 2019). Selecting teaching methods that can promote nursing students’ critical thinking is crucial. However, finding an optimal teaching method and considering different aspects can be challenging. A major issue is that training in critical thinking skills does not necessarily mean that nursing students apply this capability to clinical situations (Merisier et al., 2018). One challenge specific to simulation-based education is that the method can create anxiety in students, which can inhibit learning (Stein, 2020). Another is that simulation with high-fidelity manikins involves budget issues for universities due to the costs involved in creating, operating, and maintaining the equipment (Eliadis & Verkuyl, 2019). Unfortunately, not all faculties have the resources needed to incorporate and use high-fidelity simulation in their curricula. This form of simulation is also labor-intensive (Hung et al., 2021) and thereby time-consuming for faculty. Thus, blended simulation-based education could be a cost- and time-efficient measure for faculty.

Critical thinking is required to gather, evaluate, and manage information to decide what to do in an emergency. Making a decision is both an important and risky part of emergency situations since stress and self-doubt, limit individuals’ capability to think critically (Sterner et al., 2019; Willers et al., 2021). As a result, it is important to practice critical thinking in situations where useful decision-making strategies are applied. According to the findings in this study, blended simulation is one method of supporting nurse students’ critical thinking.

This study indicates that there were no differences in critical thinking capability based on sex or age. This is consistent with previous research involving both freshmen and senior nursing students (Azizi-Fini et al., 2015). However, the findings in previous research have been conflicting in this regard. Some research has indicated the significance of sex or age in critical thinking capability (Van Nguyen & Liu, 2021), while other research has not supported this finding (Liu et al., 2019). Zuriguel-Pérez et al. (2019) revealed that critical thinking capability is higher in senior nurses than in younger nurses. Research has also indicated that critical thinking can be developed and improved in light of other factors than age, such as clinical practice and education (Wangensteen et al., 2010). This means that what significance sex or age has on the capability to think critically can only be speculated about. Individuals may differ in their levels of critical thinking, but these differences are not necessarily linked to their sex or age. Educational and social factors might be other factors that support the development of critical thinking. Therefore, it is important to be aware that sex- and age-related stereotypes can influence perceptions of nursing students’ critical thinking capabilities.

## Strengths and Limitations

This study has certain limitations that need to be addressed. The total population had a relatively low response rate (42%) (Polit & Beck, 2016). Reasons for this limitation can only be speculated about, but all nursing students in these cohorts were affected by the COVID-19 pandemic and its consequences on education and practice. The use of a pretest–post-test study design without a control group is not the best possible design for highlighting causal effects (Polit & Beck, 2016). However, because the present course studied herein is a mandatory part of nursing education, randomization or a control group was not possible. Lastly, this study was conducted on a 3-year bachelor's nursing program. However, educational systems differ globally, meaning that the lengths of nursing programs vary, as do, for example, the diploma or bachelor's exams. The data presented in this study are self-reported measures of critical thinking and not objective measures. However, this study used a validated instrument for measuring critical thinking in nursing students, which should be considered a strength (Harper et al., 2021).

## Implications for Practice

Blended simulation-based education is an innovative teaching method for training in which nursing students can interact with nonreal patients in a realistic healthcare environment. Introducing blended simulation-based education that combines computer-based simulation and high-fidelity simulation into the nursing curriculum may contribute to the development of nursing students’ critical thinking capabilities.

## Conclusion

According to the findings of this study, a blended simulation course during nursing education can contribute to increasing nursing students’ critical thinking capabilities. Nurses’ critical thinking abilities can have an impact on patient safety, and it is thus important for nurse educators to continue to build knowledge on educational initiatives that facilitate critical thinking abilities during nursing education. As a result, this study continues to build on the use of simulation as a measure for developing and promoting critical thinking abilities during nursing education. Future research should focus on the effectiveness of using blended simulation-based education as a measure to facilitate further development of core competencies in nursing education.
